# The metabolic effect of *Momordica charantia* cannot be determined based on the available clinical evidence: a systematic review and meta-analysis of randomized clinical trials

**DOI:** 10.3389/fnut.2023.1200801

**Published:** 2024-01-11

**Authors:** Eszter Laczkó-Zöld, Boglárka Csupor-Löffler, Edina-Blanka Kolcsár, Tamás Ferenci, Monica Nan, Barbara Tóth, Dezső Csupor

**Affiliations:** ^1^Department of Pharmacognosy and Phytotherapy, "George Emil Palade" University of Medicine, Pharmacy, Sciences, and Technology of Târgu Mureş, Târgu Mureş, Romania; ^2^Institute for Translational Medicine, Szentágothai Research Centre, Medical School, University of Pécs, Pécs, Hungary; ^3^Physiological Controls Research Center, Óbuda University, Budapest, Hungary; ^4^Department of Statistics, Corvinus University of Budapest, Budapest, Hungary; ^5^Pharmacy Department, Encompass Health Rehabilitation Hospital of Round Rock, Round Rock, TX, United States; ^6^Institute of Clinical Pharmacy, University of Szeged, Szeged, Hungary; ^7^Institute of Pharmacognosy, University of Szeged, Szeged, Hungary

**Keywords:** *Momordica charantia*, bitter melon, metabolic syndrome, insulin resistance, obesity, cardiovascular disease, dyslipidemia

## Abstract

Several studies have shown that *Momordica charantia* L. (Cucurbitaceae, bitter melon) has beneficial effects on metabolic syndrome (MetS) parameters and exerts antidiabetic, anti-hyperlipidemic, and anti-obesity activities. Since the findings of these studies are contradictory, the goal of this systematic review and meta-analysis was to assess the efficacy of bitter melon in the treatment of metabolic syndrome, with special emphasis on the anti-diabetic effect. Embase, Cochrane, PubMed, and Web of Science databases were searched for randomized controlled human trials (RCTs). The meta-analysis was reported according to the PRISMA statement. The primary outcomes of the review are body weight, BMI, fasting blood glucose, glycated hemoglobin A1c, systolic blood pressure, diastolic blood pressure, serum triglyceride, HDL, LDL, and total cholesterol levels. Nine studies were included in the meta-analysis with 414 patients in total and 4–16 weeks of follow-up. In case of the meta-analysis of change scores, no significant effect could be observed for bitter melon treatment over placebo on fasting blood glucose level (MD = −0.03; 95% CI: −0.38 to 0.31; I^2^ = 34%), HbA1c level (MD = −0.12; 95% CI: −0.35 to 0.11; I^2^ = 56%), HDL (MD = −0.04; 95% CI: −0.17 to 0.09; I^2^ = 66%), LDL (MD = −0.10; 95% CI: −0.28 to 0.08; I^2^ = 37%), total cholesterol (MD = −0.04; 95% CI: −0.17 to 0.09; I^2^ = 66%,), body weight (MD = −1.00; 95% CI: −2.59–0.59; I^2^ = 97%), BMI (MD = −0.42; 95% CI: −0.99–0.14; I^2^ = 95%), systolic blood pressure (MD = 1.01; 95% CI: −1.07–3.09; I^2^ = 0%) and diastolic blood pressure levels (MD = 0.24; 95% CI: −1.04–1.53; I^2^ = 0%). *Momordica* treatment was not associated with a notable change in ALT, AST, and creatinine levels compared to the placebo, which supports the safety of this plant. However, the power was overall low and the meta-analyzed studies were also too short to reliably detect long-term metabolic effects. This highlights the need for additional research into this plant in carefully planned clinical trials of longer duration.

## 1 Introduction

Metabolic syndrome (MetS) has been defined as a complex group of risk factors for cardiovascular disease and diabetes. These factors include elevated blood pressure (systolic blood pressure ≥ 130 mm Hg, diastolic blood pressure ≥ 85 mmHg), elevated triglyceride levels (TG; ≥150 mg/dL), low high-density lipoprotein cholesterol levels (HDL-C; male <40 mg/dL, female <50 mg/dL), hyperglycemia (fasting blood glucose ≥100 mg/dL), and obesity (waist circumference: male ≥94 cm, female ≥80 cm) (1, 2). It has been reported that approximately 30% of the world population is affected by MetS, making it a major global health challenge, and an important cause of mortality and morbidity (3, 4). The treatment of MetS is based on an improvement of lifestyle, promoting physical activity, and a balanced low-energy diet (2). Some medicinal plants may be useful tools in the treatment of several MetS components before beginning pharmacological therapy or to supplement medical treatment (2, 5).

Diabetes is one of the most common metabolic diseases and its prevalence is increasing steadily worldwide. According to International Diabetes Federation (IDF) data published in 2021, over 530 million adults are living with diabetes worldwide, and by 2045 their number will exceed 783 million (6). Direct complications from diabetes can lead to heart attack, stroke, blindness, kidney failure, and lower limb amputation (7). Many plants used in folk medicine were involved in clinical trials for assessing their potential as antidiabetic agents and for their positive effect on the treatment or prevention of MetS (2, 8–11). Among these are *Aloe vera* (L.) Burm.f. (10, 11), *Capparis spinosa* L. (12), *Cinnamomum cassia* and *C. zeylanicum* (13, 14), *Trigonella foenum-graecum* (15), *Coffea arabica, Theobroma cacao* (16), *Allium sativum* (2)*, Gymnema sylvestre* (17, 18), *Curcuma longa* (19, 20), *Thea sinensis* (21), *Ilex paraguariensis* (22) and *Momordica charantia* (23).

*Momordica charantia* L. (Cucurbitaceae, bitter gourd or bitter melon) is a tropical and subtropical vine, the edible fruits, shoots, and leaves of which are widely used in the East Asian, South Asian, and Southeast Asian cuisines. Various parts of the plant, especially the fruits, are used in folk medicine in Asia and Africa. The medicinal use of the plant is preponderant in the treatment of diabetes. Various preclinical and clinical studies conducted so far showed the protective effects of *M. charantia* against metabolic syndrome and its associated disorders. The bitter gourd extracts were evaluated for numerous pharmacological activities, and most of them were performed on animals. Fruits and seed extracts reduced fasting glucose and glycosylated hemoglobin A1c in comparison to vehicle control when tested in animal models of type 2 diabetes (24). The water extract of leaves proved to have an anti-obesity effect on a high-fat diet (HFD)-induced obese mouse model through regulating lipid metabolism (25). Hypoglycemic and hypolipidemic effects of different fruit parts were tested on normal, hyperglycemic, and hyperlipidemic rats (26, 27).

The mechanism of action of *M. charantia* has not been fully elucidated. Charantin, a steroidal saponin mixture isolated from the plant is its main active constituent. Charantin has been shown to have insulin-like activity by augmenting insulin release, reducing gluconeogenesis, increasing hepatic glycogen synthesis, and increasing peripheral glucose oxidation (28). Antidiabetic activity could be confirmed for charantin, but not for steroidal saponin aglycones (29). The charantin-rich fraction of *M.charantia* reduced blood sugar levels in type 1 and type 2 diabetic animal models (30, 31). The treatment with the *M. charantia* extracts decreased plasma insulin and increased insulin sensitivity by increasing the expression of GLUT4 in the skeletal muscle and of IRS-1 in the liver of mice with type 2 diabetes. However, no effect on insulin sensitivity was detected in mice with type 2 diabetes (28).

Certain clinical trials suggested that bitter melon products may be promising phytomedicines to manage hyperglycemia (32, 33), ameliorating systemic complications of type 2 diabetes (33), including associated cardiovascular risk factors (34) and it can be considered in obesity management too (35, 36). In the last five years, numerous review articles have been published about bitter melon chemical compounds (37, 38), nutritional value (39, 40), and pharmacological actions (36, 41–43), but only a few are with meta-analysis. Jandari et al. performed a systematic review and meta-analysis of randomized clinical trials regarding the effect of bitter melon on blood pressure (44), Cortez-Navarrete et al. reviewed the metabolic effects of bitter melon reported in clinical trials (45), but without performing a meta-analysis, while Peter et al. evaluated the efficacy in lowering the elevated plasma glucose level in diabetes mellitus (46). The present systematic review and meta-analysis aimed to evaluate the effect of bitter melon on metabolic syndrome parameters.

## 2 Methods

### 2.1 Population, intervention, comparison, outcomes, and study design

The following PICO (patients, intervention, comparison, outcome) format was applied: P: patients in prediabetes or diagnosed with type 2 diabetes; I: *Momordica*; C: placebo; and O: change in metabolic parameters. We used PRISMA statement (47) to report the meta-analysis results.

### 2.2 Systematic review protocol

This systematic review and meta-analysis were registered in the International Prospective Register of Systematic Reviews (PROSPERO) *a priori* (ID: CRD42021293139).

### 2.3 Search strategy and data sources

We systematically searched PubMed/MEDLINE, Embase, Cochrane, and Web of Science databases for articles reporting randomized, parallel-group, placebo-controlled clinical trials up to October 31, 2023, without limiting the language or publication year. The following main keywords and related terms were used: “momordica” AND “diabetes.” For transparency purposes this meta-analysis relied on publicly available data. There was no need to contact the authors of the articles nor the manufacturers of the products under consideration for any additional information. We removed duplicate and records lacking an abstract and the final selection was based on article titles and abstracts. Two reviewers (EBK, BCL) independently reviewed the full texts of the remaining records. Any disagreement among reviewers was discussed and resolved, a third reviewer was available for consultation at any time (DC). We used Mendeley (version 1.19.8; Mendeley Ltd.) to manage references.

### 2.4 Study selection

Only randomized, placebo-controlled studies with adult prediabetes or with a type 2 diabetes mellitus diagnosis were included in the meta-analysis. A minimal follow-up duration of 4 weeks was determined as an inclusion criterion since this is the minimum period required for meaningful effects on glucose control as assessed by HbA1c concentration (48). Case series, case reports, non-randomized and open-label studies, and trials performed with patients with concomitant diseases affecting blood glucose levels or in which the intervention contained other active ingredients than *M. charantia* were not considered for analysis.

### 2.5 Data extraction

Data collection was executed following the PRISMA guidelines. The two independent reviewers (EBK, DC) extracted study characteristics and results. Any discrepancies in the extracted data were discussed and resolved. The following data items were selected from the included papers: study design, sample size and characteristics of the patient population, duration, intervention details, body weight, BMI, waist circumference, body fat, systolic and diastolic blood pressure, HbA1c, fasting glucose, total cholesterol, triglyceride, HDL, LDL, VLDL, creatinine, ALT, and AST levels. A statistical analysis of at least three clinical trials involving different patient populations was required for each outcome.

### 2.6 Statistical analysis

Papers included in this meta-analysis reported data in three ways:

- a pre-intervention value and a change score,
- a pre- and post-intervention value,
- a pre- and post-intervention value along with a change score.

Two distinct types of analysis were undertaken:

- Analysis of post-intervention values only (without using baseline or change information); this is unbiased in the case of randomized controlled trials, but is inefficient, however, the loss of efficiency is marginal if the correlation between the pre- and post-intervention values is less than 0.5 (49).
- Analysis of change scores; this is more efficient, especially if the correlation between the pre- and post-intervention values is higher than 0.5.

The first analysis requires the imputation of the post-intervention value in scenario A, the second requires the imputation of the change score in scenario B (no value must be imputed in all other combinations). Both imputation task requires the knowledge of correlation between the pre- and post-intervention values, this was obtained from studies of type C, i.e., studies with all data given were used to calculate the correlation which was then used to impute studies with partial information. The correlation coefficient was calculated as the sum of the variances of pre- and post-intervention values minus the variance of the change divided by two times the product of the standard deviation of the pre- and post-intervention values (50, 51). This was calculated separately for all studies, arms (i.e., placebo or active), and outcomes, and were then averaged across studies, i.e., an average was calculated for each outcome and arm after discarding impossible values (i.e., a correlation larger than 1 in absolute value). This average was used for imputation only if the range of the correlations for the given outcome and arm was less than 0.4, otherwise that outcome and arm’s correlation was not imputed. After the value used for imputation was obtained, the post-intervention values were imputed using scenario A, and the change scores were imputed for scenario B.

Both types of meta-analysis (post-intervention and change score) were then run using the imputed datasets. The outcome measure was the mean difference. In case when median and lower/upper quartile was given, mean and standard deviation was estimated with the quantile estimation method of McGrath et al. (52). Common-effect meta-analysis, and random-effects meta-analysis (with restricted maximum likelihood estimation) was carried out (53).

Results are presented using standard forest plots, depicting both common- and random-effects results, together with the usual τ^2^ and I^2^ heterogeneity statistics and a test for the overall effect (both for the random-effects results).

Calculations were conducted under R statistical environment version 4.2.1 (54) using package metafor version 3.8–1 (55). Full source code is available at https://github.com/tamas-ferenci/MomordicaMetaAnalysis.

### 2.7 Risk of bias analysis

The Cochrane Collaboration tool was used to assess the risk of bias, which includes seven specific domains: random sequence generation, allocation concealment, blinding of participants and personnel, blinding of outcome assessment, incomplete outcome data, selective reporting, and other bias scores. Studies were classified as having a high (red), unclear (yellow), or low (green) risk of bias in each domain. Disagreements about the quality of the studies were settled through discussion (TF, DC). The risk of bias summary table and figure were generated by the RevMan 5 software (56).

### 2.8 Ethics statement

Ethical approval was not needed because we only collect non-confidential information from which the patients’ identities cannot be determined.

## 3 Results

### 3.1 Search results

**Figure 1:**
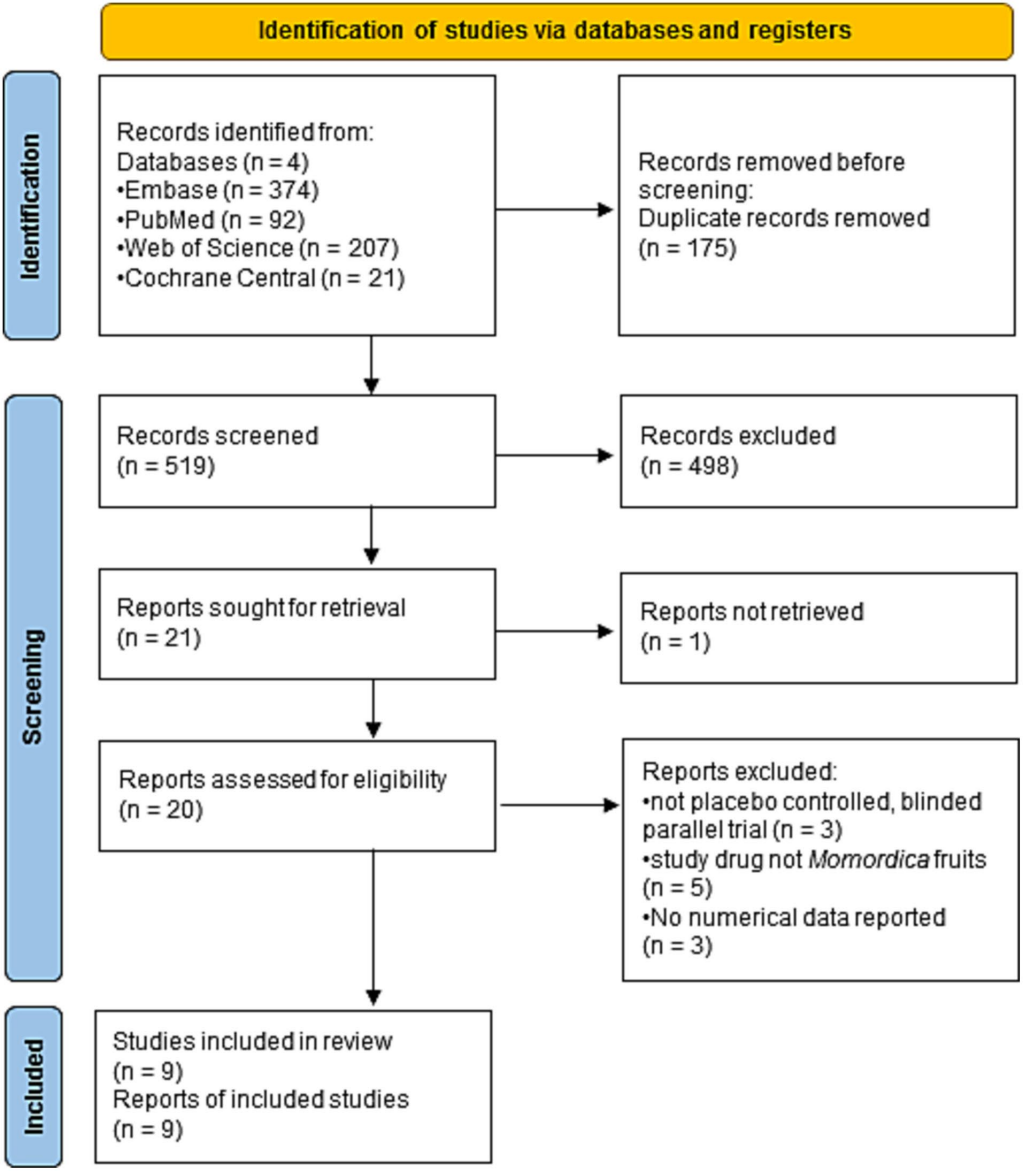
Flow diagram of study identification and selection by PRISMA 2020.

The search resulted in 694 hits, after removing the duplicates 519 records remained and were potentially eligible for inclusion. In the full-text section stage, most articles were excluded due to the lack of placebo control (57, 58), lack of blinding (59), lack of numerically reported results (32, 60, 61), and since other plant parts (leaves) than *M. charantia* fruits were used as study (59, 62–65). We identified nine trials eligible for our review and meta-analysis (33, 66–73). The selection process is presented in Figure 1 (47).

### 3.2 Study characteristics

We analyzed nine randomized placebo-controlled trials (33, 66–73). Table 1 contains the summary of the main study and patient characteristics. We included only randomized double or single-blinded studies released between 2003 and 2023. Studies length varied between 4 and 16 weeks, the sample size was low (24–90 participants), the study drugs were inconsistent in quality (dry plant material and dry extracts) and in quantity (the daily dose was 2–6 g fruit or extract of fruit equivalent to 9 g fruit). The total number of participants was 414 and the locations of the studies were different Asian or North American countries. The minimum age of participants was 18 years (67) or 20 years (33), and the oldest patient was 80 years old (71), according to the available information.

**Table 1:** Baseline characteristics of studies included in the meta-analysis (RCT: randomized, controlled trial; DB: double blind; SB: single-blind).

**Article**	**Country**	**Study design**	**Participants**	**Duration**	**Study drug**	**Daily dose**
Cortez-Navarrete, 2018	Mexico	RCT, DB	24	12 weeks	dried powder of the fruit pulp	2 g
Cortez-Navarrete, 2022	Mexico	RCT, DB	24	12 weeks	Commercial herbal supplement *	4 capsules (2000 mg)
Dans, 2007	Republic of the Philippines	RCT, DB	40	13 weeks	special extract from fruits and seeds	2 capsules
John, 2003	India	RCT, SB	50	4 weeks	dried fruit	6 g
Kim, 2020	Republic of Korea	RCT, DB	90	12 weeks	dry fruit extract	2.38 g
Kim, 2023	Republic of Korea	RCT, DB	65	12 weeks	dry fruit extract	2.4 g
Kinoshita, 2018	Japan	RCT, DB	43	30 days	dry fruit extract	300 mg extract (equivalent to 9 g fruit)
Trakoon-osot, 2013	Thailand	RCT, DB	38	16 weeks	dried pulp	6 g
Yang, 2022	Taiwan	RCT, DB	40	3 months	mcIRBP-19^**^ containing extract	600 mg

*500 mg capsules, each contains 200 mg Momordica extract (standardized to 2.5% bitter principle) and 300 mg dried fruit powder.

**Momordica charantia insulin receptor binding peptid-19.

### 3.3 Risk of bias assessment

Overall, the methodical quality of the trials included in our final quantitative analysis was reckoned to be good, only with low or unclear risk of bias for double-blind randomized trials (Supplementary Figures S1, S2).

Random sequence generation was described in six studies (33, 67–70, 72); however, the measures taken to ensure allocation concealment were given in only one trial (67). Performance and detection biases were unclear in five studies (33, 68–71) because the authors of these studies failed to report whether the intervention and the comparator were identical in size, shape, color, and odor; and it remained unclear whether the outcomes were assessed in a blinded manner or not. The study of Cortez-Navarrante (72) was judged to have low risk of performance and detection biases, because based on their article nor the patients neither the investigators were aware of the assigned treatment. Dans et al. stated that the treatment and the placebo capsules were identical, and the patients, the investigators, and the statistician were unaware of the treatments received until the end of the statistical analysis. Therefore, this study had low risk of performance and detection biases (67). However, in the study of John et al. (66) the investigators were not blinded and the tablets were dissimilar; therefore, this study had high risk of performance and detection biases.

All the included studies showed a minimal risk of attrition and reporting biases. The study of Dans et al. was funded by a company, but the authors stated that the sponsor had had no role in the data collection or the analysis of the study; therefore, the risk of other bias in this study remained low (67). However, in the study of John et al., the investigators were not blinded, and the tablets were dissimilar; therefore, this study had a substantial risk of performance bias (66).

### 3.4 Main findings

#### 3.4.1 Effect of *Momordica charantia* fruits on glycemic indices

**Figure 2:**
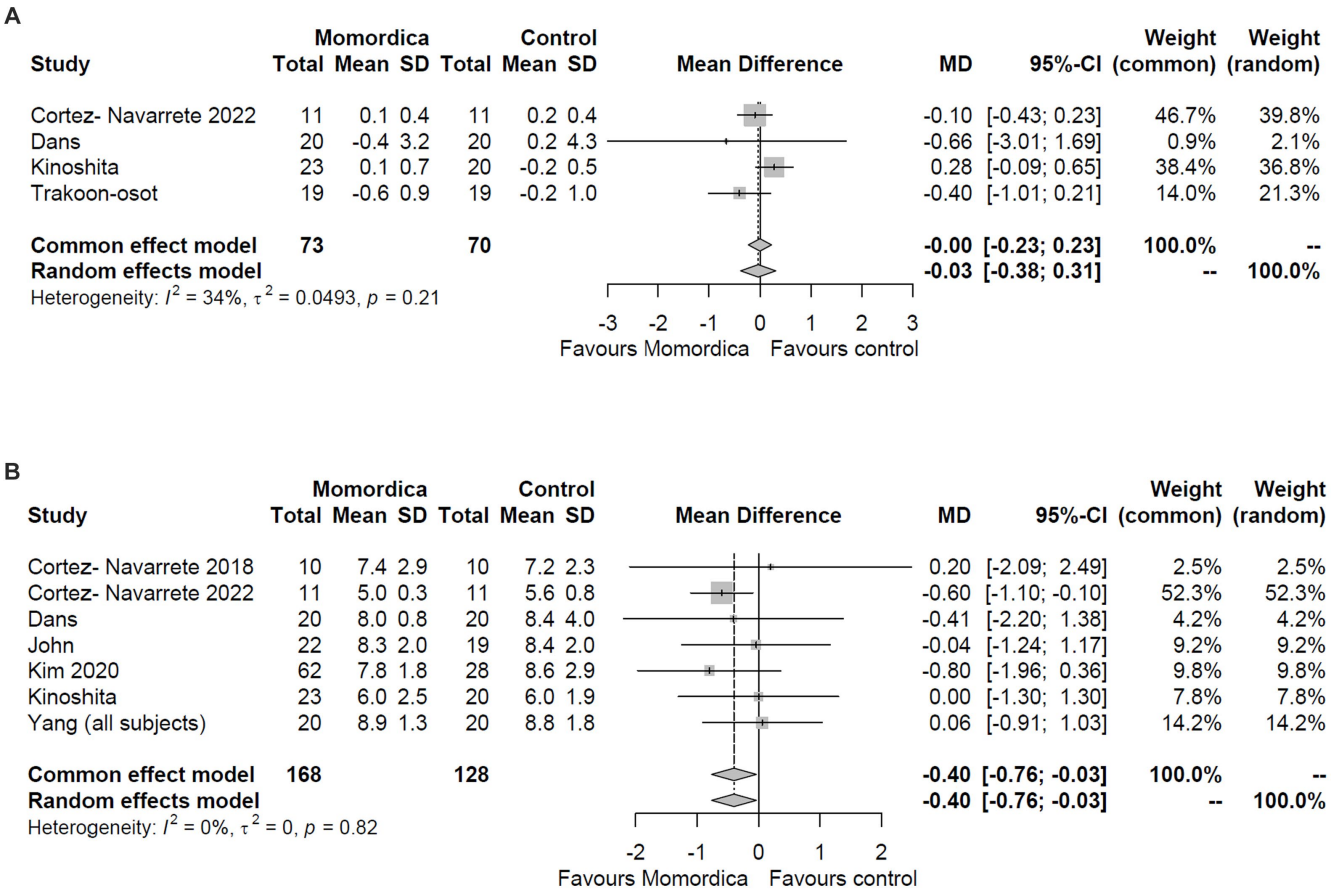
The effect of *Momordica charantia* on blood glucose level compared to placebo in the meta-analyses of the change scores **(A)** and post-intervention values **(B)** using the random effects and common effect models.

One primary outcome was fasting blood glucose level and relevant data were available from eight trials (Figure 2). Using the random effects model, the superiority of *M. charantia* over placebo on fasting blood glucose level could not be observed (MD = −0.03; 95% CI: −0.38 to 0.31; I^2^ = 34%) with the analysis of change scores (Figure 2A). This finding was consistent when analyzing the data with the common-effect model. However, the assessment of the post-intervention suggests that *M. charantia* is more effective than placebo in decreasing fasting blood glucose levels (MD = −0.40; 95% CI: −0.76 to −0.03; I^2^ = 0%) (Figure 2B). The primary reason of this contradiction is that the first analysis was based on 4 trials only (143 participants), whereas post-intervention values were available in 7 trials (296 participants) and missing values could not be imputed, because the calculated correlations were unacceptably dissimilar.

**Figure 3:**
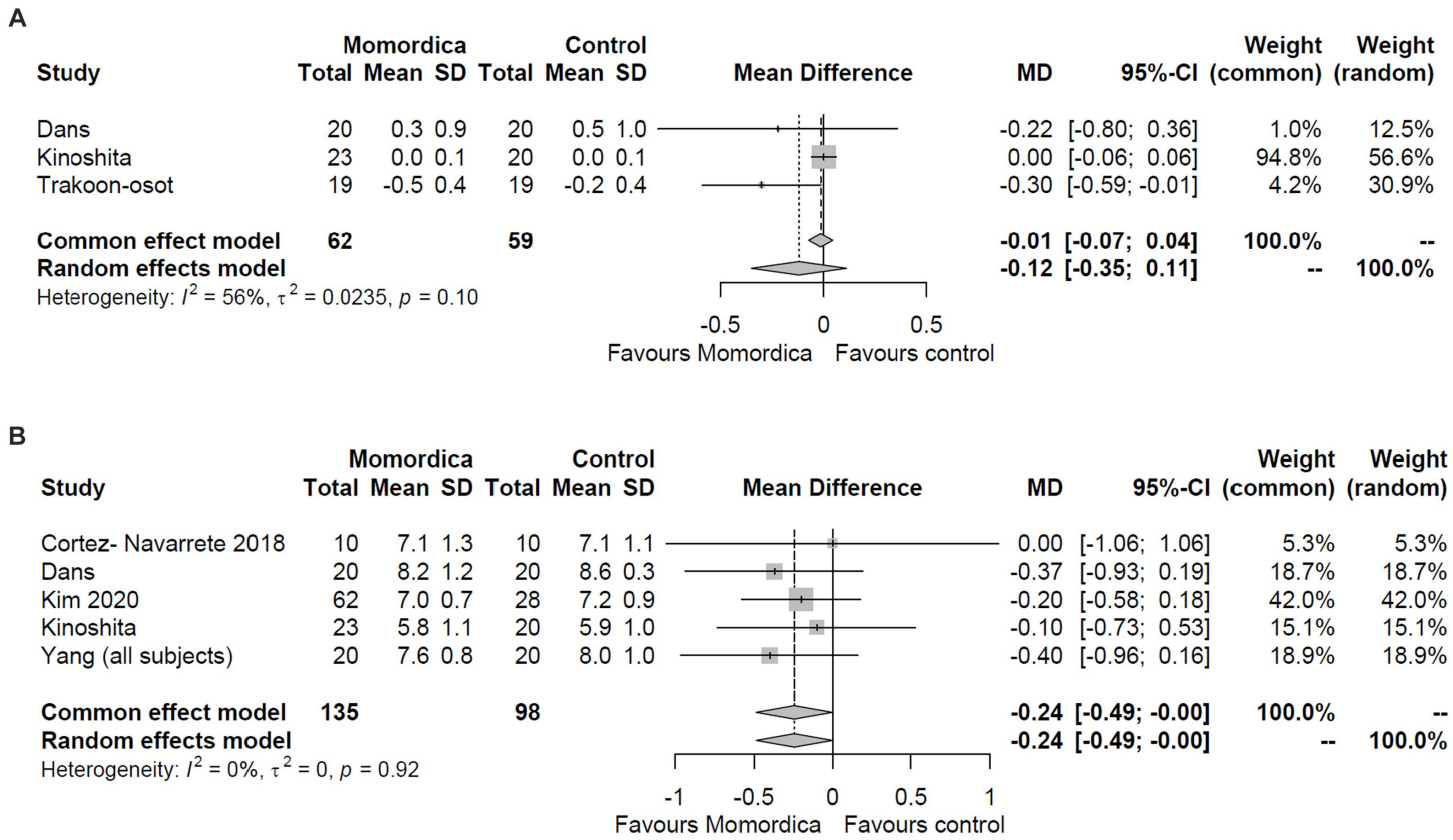
The effect of *Momordica charantia* on HbA1C level compared to placebo in the meta-analyses of the change scores **(A)** and post-intervention values **(B)** using the random effects and common effect models.

The analysis of HbA1c level resulted in somewhat similar results (Figure 3). The analysis of change scores was based on three studies (with data from 121 patients), whereas the analysis of post-intervention values on five trials (with data from 233 patients), and again, values could not be imputed. The meta-analysis of the post-intervention values using the random effects model indicated borderline efficacy for the *M. charantia* treatment with an MD = −0.24 (95% CI, −0.49 to 0.00, I^2^ = 0%) (Figure 3B), however, the analysis of the changes scores definitely did not show superiority over placebo (MD = −0.12; 95% CI: −0.35 to 0.11; I^2^ = 56%) (Figure 3A).

One of the recent trials (73) includes clinical indicators to assess insulin sensitivity and glucose metabolism, like HOMA-IR, Matsuda index, insulinogenic index. Because these parameters were not found in the other studies, they could not be evaluated in the meta-analysis.

#### 3.4.2 Effect of *Momordica charantia* fruits on lipid profile

**Figure 4:**
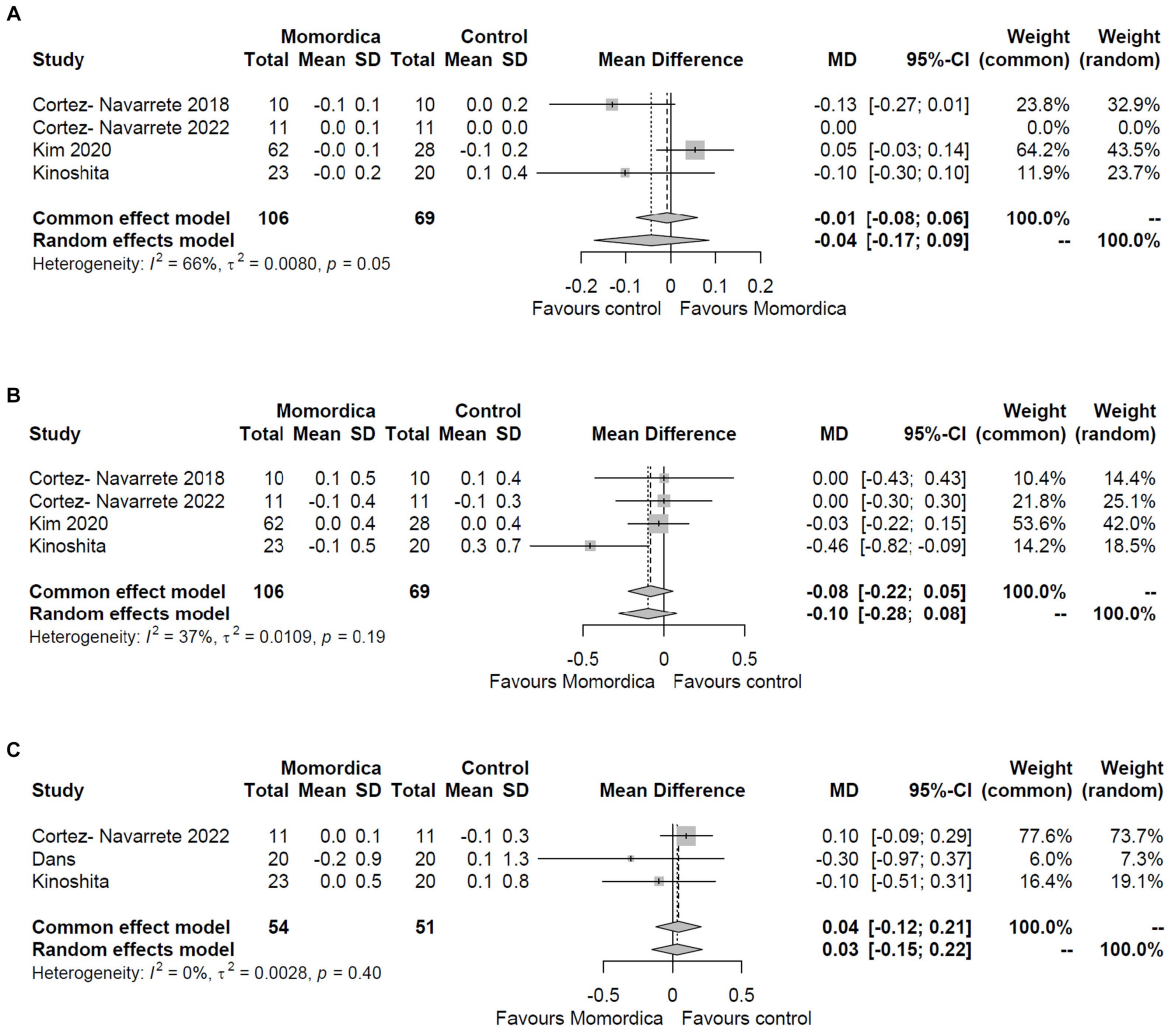
The effect of *Momordica charantia* on HDL **(A)**, LDL **(B)**, and total cholesterol levels **(C)** compared to placebo in the meta-analyses of the change scores using the random effects and common effect models.

Regarding serum lipid levels, only the effects on HDL, LDL, and total cholesterol levels could be analyzed. Based on the changes in the mean differences data, as reported in three or four RCTs (Figure 4), *M. charantia* was not superior in any of these outcomes compared to placebo using the random effects model (MD = −0.04; 95% CI: −0.17 to 0.09; I^2^ = 66%; MD = −0.10; 95% CI: −0.28 to 0.08; I^2^ = 37%; and MD = −0.03; 95% CI: −0.15 to 0.22; I^2^ = 0%, respectively). These results are consistent with those obtained using common effect model.

The assessment of the post-intervention values complemented these results with triglyceride data. There was no evidence of significant effects of *M. charantia* on HDL (MD = −0.02; 95% CI: −0.12 to 0.09; I^2^ = 0%), LDL (MD = −0.01; 95% CI: −0.22 to 0.19; I^2^ = 0%), total cholesterol (MD = −0.04; 95% CI: −0.27 to 0.19; I^2^ = 0%) and triglyceride levels (MD = −0.09; 95% CI: −0.39 to 0.20; I^2^ = 22%) (Supplementary Figure S3). The reliability of these results is reassured by low heterogeneity values and the consistency of common and random effects models.

The assessment of the VLDL data is not conclusive, as data from only two studies were available (68, 72).

#### 3.4.3 Effect of *Momordica charantia* fruits on anthropometric parameters

No significant effect on body weight (MD = −1.00; 95% CI: −2.59 to 0.59; I^2^ = 97%), body fat (MD = −1.21; 95% CI: −2.62 to 0.20; I^2^ = 0%) and BMI (MD = −0.42; 95% CI: −0.99 to 0.14; I^2^ = 95%) was observed in the meta-analyses of change scores. The analysis of the post-intervention values yielded the same results (Supplementary Figure S4). The effect on waist circumference could only be assessed from post-intervention data (MD = 0.72; 95% CI: −3.36 to 4.80; I^2^ = 13%) (Supplementary Figure S4). Common and random effects models had consistent results.

#### 3.4.4 Effect of *Momordica charantia* fruits on blood pressure

Administration of *M. charantia* fruits did not demonstrate any effect on systolic blood pressure (MD = 1.01; 95% CI: −1.07 to 3.09; I^2^ = 0%) or diastolic blood pressures (MD = 0.24; 95% CI: −1.04 to 1.53; I^2^ = 0%) when comparing the change scores to that of the placebo group. This is supported by the examination of the post-intervention data (Supplementary Figure S5). Heterogeneity was very low and common and random effects model had similar results.

### 3.5 Adverse effects

Overall, there were no serious adverse effects reported by the studies included in our meta-analysis. Headache and gastrointestinal complaints were the most reported adverse events. In a double-blind RCT that used a special extract from fruits and seeds of *M. charantia* in addition to standard antidiabetic medication, adverse effects such as diarrhea and epigastric pain were reported after 1-month of administration (67).

The consumption of 6 g bitter melon pulp per day resulted in a significantly higher frequency of diarrhea and flatulence than in the placebo group (33).

Based on data from four trials (33, 67, 68, 72), *M. charantia* administration exerted no significant effects on liver enzymes (ALT, AST) and creatinine levels (Supplementary Figure S6) compared to placebo, however confidence intervals were sometimes wide to draw reliable safety conclusions (i.e., power was low).

## 4 Discussion

### 4.1 Main findings

A vast number of studies have been conducted, in both animal and human subjects using the *M. charantia* plant or different extracts prepared with stems, leaves, and fruits of the plant. These studies have allowed the identification of a few health-promoting benefits, including hypolipidemic, hypoglycemic, and anti-obesity effects (25, 26, 32, 33, 35, 36). In this review and meta-analysis, we have systematically evaluated the existing evidence on the potential efficacy of bitter melon in the treatment of metabolic syndrome, based on randomized, parallel-group, placebo-controlled trials. All the included trials, except the studies of Cortez-Navarrete (68, 72) were carried out in different Asian countries. Based on the nine trials included in this study, our findings show that *M. charantia* mono herbal preparations do not have a significant overall positive influence on blood glucose levels and other cardiovascular risk factors associated with metabolic syndrome. However, *M. charantia* was found to be statistically significantly more effective than a placebo in terms of reducing HbA1C and (marginally) fasting glucose levels when post-intervention data were analyzed. Even this relatively week conclusion (*p* = 0.032 and *p* = 0.050 respectively) was dependent on the analytical approach used, as it vanished when change scores were used. The change score data set had smaller sample size and was much more heterogeneous. Overall, our confidence in this finding is therefore low. The absence of an impact on ALT, AST, and creatinine levels suggests that there were no potential hepato- or nephrotoxic consequences at the doses used, although, the small sample size limits power and does not allow the reliable assessment of safety.

A previous meta-analysis suggested that bitter melon alone or in combination with other herbal medicinal products can reduce the elevated fasting plasma glucose level (FPG), postprandial glucose (PPG), and glycated hemoglobin A1c (HbA1c) (46). Compared to the placebo, *M. charantia* significantly reduced FPG, PPG, and HBA1c with mean differences of −0.72 mmol/L, − 1.43 mmol/L, and − 0.26%, respectively. *M. charantia* also lowered FPG in prediabetes (mean difference − 0.31 mmol/L). As discussed above, our meta-analysis found only a very week evidence for the superiority over placebo when assessing the effect on blood glucose and HbA1c levels. The explanation for this discrepancy is that the dataset used for analysis was different. First, we included five trials that were published after the previous meta-analysis (69–73). Second, the positive outcome of the meta-analysis of Peter et al. (46) could be explained by the inclusion of a paper reporting a trial with a size effect in favor of *M. charantia* (58) that was excluded in the present investigation due to the lack of blinding.

Animal experiments suggest that *M. charantia* might have the potential for increasing insulin sensitivity in patients with type 2 diabetes (28). According to a recent meta-analysis of animal experiments with type 2 diabetic rats, fruit and seed extracts of *M. charantia* reduced fasting plasma glucose and after at least 3 months of treatment increased serum insulin level and reduced HbA1c, triglycerides, total cholesterol in comparison to vehicle control (24). However, it should be noted that although the dose of *M. charantia* applied in different experiments was diverse, the typical range of 150–600 mg dry extract/kg/day is several magnitudes higher than those in the clinical trials. These differences in dosing might be one explanation for the lack of efficacy observed in this meta-analysis. Furthermore, differences in the qualitative and quantitative composition may contribute to the outwardly unreliable efficacy. Although charantin is considered the major active constituent of the plant, *in silico* studies suggest the importance of further metabolites. Momordicoside D (ligand of Takeda-G-protein-receptor-5, TGR5), cucurbitacin (ligand of glucagon-like peptide-1 receptor, GLP-1r), and charantin (ligand of dipeptidyl peptidase 4, DPP-4) were identified as the antidiabetic constituents of bitter melon *in silico*. In subsequent animal experiments, these potential mechanisms of action were reassured, since the extract of *M. charantia* significantly increased the expression of GLP-1r and TGR5 and decreased the expression of DPP-4 (74). The complex mechanism of action and the presence of multiple active components in this plant urge the need for the standardization of clinically studied products.

The lack of unambiguous efficacy on blood metabolic parameters might also be due to the short duration of the studies. HbA1c level reflects the cumulative glycemic history of the preceding two to three months (75). In the case of lipid levels, the efficacy of lifestyle changes can be expected within 3–6 months, and even in the case of a statin or combined therapy, the maximum percentage change will occur by 4 to 12 weeks after starting (76). Some of the studies meta-analyzed by us were only 30 days long (66, 69).

Regarding the effect on blood pressure, our findings are in accordance with the meta-analysis performed by Jandari et al. (44), which concluded that bitter melon preparations do not exert a significant antihypertensive effect. The effect of *M. charantia* on systolic and diastolic blood pressure was investigated in five trials (including 163 participants). The pooled effect size showed that neither systolic nor diastolic blood pressure changed following *M. charantia* supplementation. *M. charantia* seemed to be more effective in younger adults or when consumed for short durations, however, none of the subgroup analyses revealed significant efficacy compared to placebo. However, the duration of the included studies was 4–16 weeks, which does not allow the assessment of long-term antihypertensive effects (44). The potential long-term effect on blood pressure might be the result of the beneficial effect on blood lipid levels. For the effects on HDL, LDL triglyceride, total cholesterol levels, and ALT, AST, and creatinine concentrations, our meta-analysis is the first independent assessment of previously published clinical data. Our results do not support the hypothesis that the impact of bitter gourd on blood lipid levels might lead to antihypertensive effect.

The strength of our study is that we included only blinded, placebo-controlled studies that assessed the effect of *M. charantia* for at least four weeks on prediabetic and diabetic patients. The analyzed studies were performed by different research groups in different countries. By excluding complex preparations, we aimed to assess the effect of this herbal component only.

### 4.2 Limitations

The most important limitation of our meta-analysis is that the number of included studies and the number of patients is low, leading to even a meta-analysis being underpowered, moreover, the applied doses were not uniform. Although we did not find unambiguous efficacy in any of the analyzed outcomes, bitter gourd was found to be effective in some clinical trials. The duration of the studies (4–16 weeks) was too short to reveal the potential effects of *M. charantia* on metabolic parameters. This highlights the need for additional research into this plant in carefully planned clinical trials.

## 5 Conclusion

Although bitter melon has been widely used by patients suffering from metabolic syndrome, the meta-analysis of randomized, placebo-controlled trials does clearly not support the rationale of this practice. In agreement with a previous meta-analysis, we did not find an effect on blood pressure. In contrast with the meta-analysis of Peter et al., our assessment did not reveal an unambiguous effect on the blood glucose level. The effect on HDL, LDL triglyceride, and total cholesterol levels was meta-analyzed for the first time by us, and we did not find any significant beneficial effect in any of the parameters. However, lack of efficacy in the short-term studies does not necessarily mean the lack of efficacy in the case of long-term treatment *M. charantia* use. The limited sample size should also be considered when interpreting this finding.

To assess the clinical efficacy of *M. charantia*, there is a call for long-term randomized controlled trials with larger sample sizes. The investigation of the dose-dependence of antidiabetic activity in humans should also be considered.

## Data availability statement

The datasets presented in this study can be found in online repositories. The names of the repository/repositories and accession number(s) can be found at: https://github.com/tamas-ferenci/MomordicaMetaAnalysis.

## Author contributions

EL-Z and DC: conceptualization. EL-Z, BC-L, and DC: methodology. E-BK and DC: data extraction and abstracts screening. E-BK and BC-L: full texts screening. TF: statistical analysis. EL-Z, MN, and DC: risk of bias analysis. EL-Z and MN: writing—original draft preparation. BC-L, TF, and DC: writing—review and editing. BT: risk of bias analysis and writing—review and editing. All authors contributed to the article and approved the submitted version.
