# Supplementary material for: The metabolic effect of Momordica charantia cannot be determined based on the available clinical evidence: a systematic review and meta-analysis of randomized clinical trials
Source: Front Nutr. 2024 Jan 11;10:1200801. doi: 10.3389/fnut.2023.1200801 (PMC10808600; doi:10.3389/fnut.2023.1200801)
Supplement: Supplementary file 1 [file Data_Sheet_1.pdf]

Supplementary Material

The metabolic effect of *Momordica charantia* cannot be determined based on the available clinical evidence: a systematic review and meta-analysis of randomized clinical trials

Eszter Laczkó-Zöld*, Boglárka Csupor-Löffler, Edina-Blanka Kolcsár, Tamás Ferenci, Monica Nan, Barbara Tóth and Dezső Csupor

*** Correspondence:** Eszter Laczkó Zöld: eszter.laczko@umfst.ro

1 Supplementary Figures

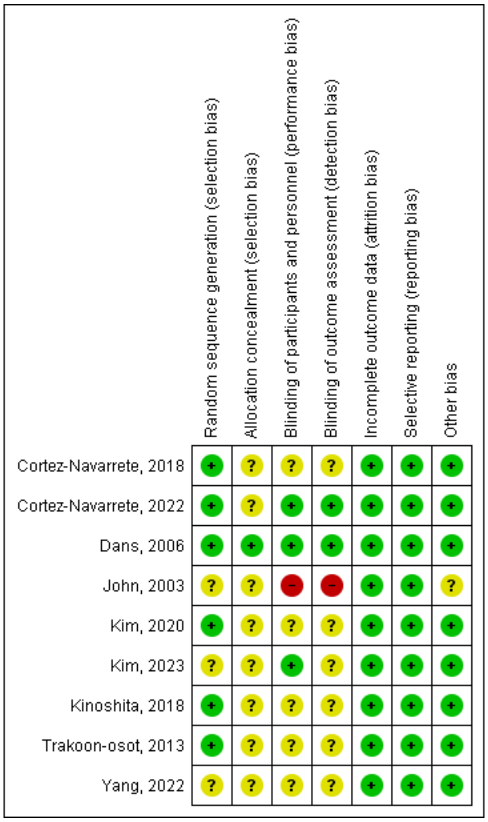

Supplementary Figure 1S. Risk of bias summary: review of authors' judgement on each risk of bias item for each included study.

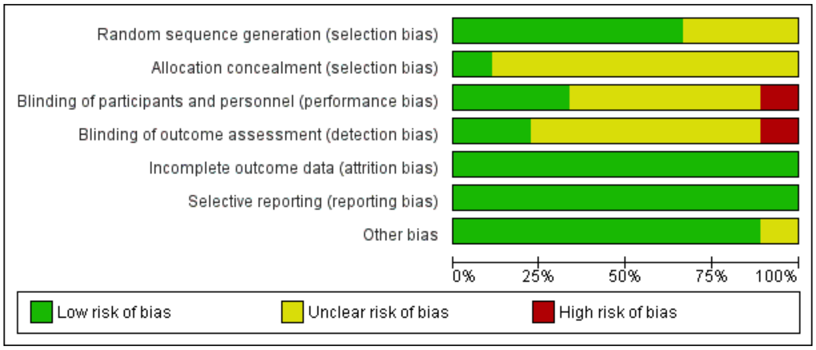

Supplementary Figure 2S. Risk of bias graph: review of authors' judgement on each risk of bias item, presented as percentages across all included studies.

A

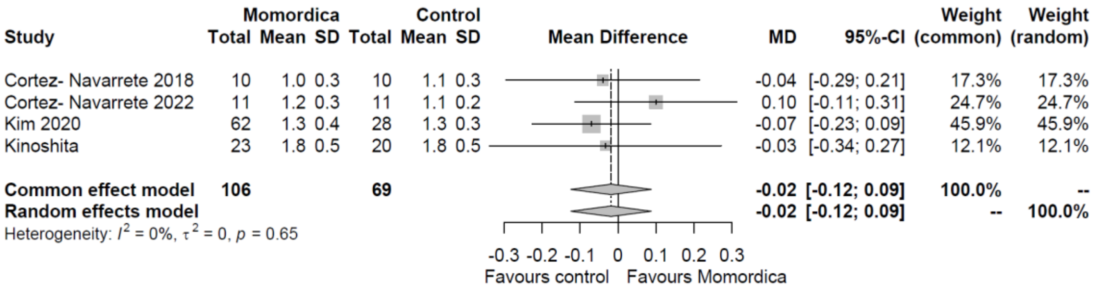
Study
Momordica Total Mean SD
Control Total Mean SD
Mean Difference
MD 95%-CI
Weight (common)
Weight (random)
Cortez- Navarrete 2018 10 1.0 0.3 10 1.1 0.3 -0.04 [-0.29; 0.21] 17.3% 17.3%
Cortez- Navarrete 2022 11 1.2 0.3 11 1.1 0.2 0.10 [-0.11; 0.31] 24.7% 24.7%
Kim 2020 62 1.3 0.4 28 1.3 0.3 -0.07 [-0.23; 0.09] 45.9% 45.9%
Kinoshita 23 1.8 0.5 20 1.8 0.5 -0.03 [-0.34; 0.27] 12.1% 12.1%
Common effect model 106 69 -0.02 [-0.12; 0.09] 100.0% --
Random effects model -0.02 [-0.12; 0.09] -- 100.0%
Heterogeneity: I2 = 0%, τ2 = 0, p = 0.65
-0.3 -0.2 -0.1 0 0.1 0.2 0.3
Favours control Favours Momordica

B

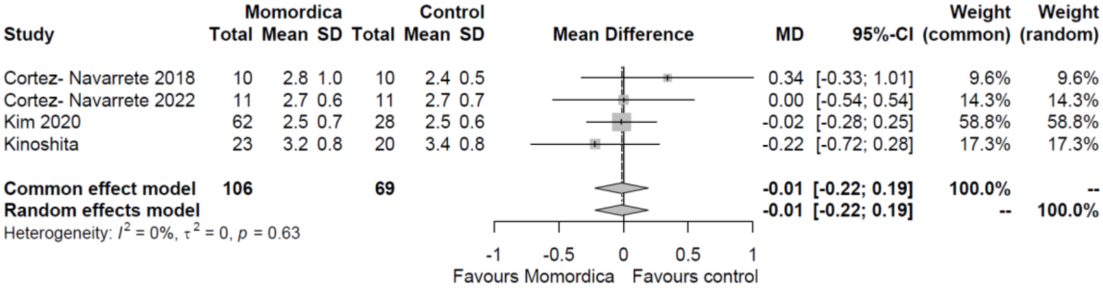
Study
Momordica Total Mean SD
Control Total Mean SD
Mean Difference
MD 95%-CI
Weight (common)
Weight (random)
Cortez- Navarrete 2018 10 2.8 1.0 10 2.4 0.5 0.34 [-0.33; 1.01] 9.6% 9.6%
Cortez- Navarrete 2022 11 2.7 0.6 11 2.7 0.7 0.00 [-0.54; 0.54] 14.3% 14.3%
Kim 2020 62 2.5 0.7 28 2.5 0.6 -0.02 [-0.28; 0.25] 58.8% 58.8%
Kinoshita 23 3.2 0.8 20 3.4 0.8 -0.22 [-0.72; 0.28] 17.3% 17.3%
Common effect model 106 69 -0.01 [-0.22; 0.19] 100.0% --
Random effects model -0.01 [-0.22; 0.19] -- 100.0%
Heterogeneity: I2 = 0%, τ2 = 0, p = 0.63
-1 -0.5 0 0.5 1
Favours Momordica Favours control

C

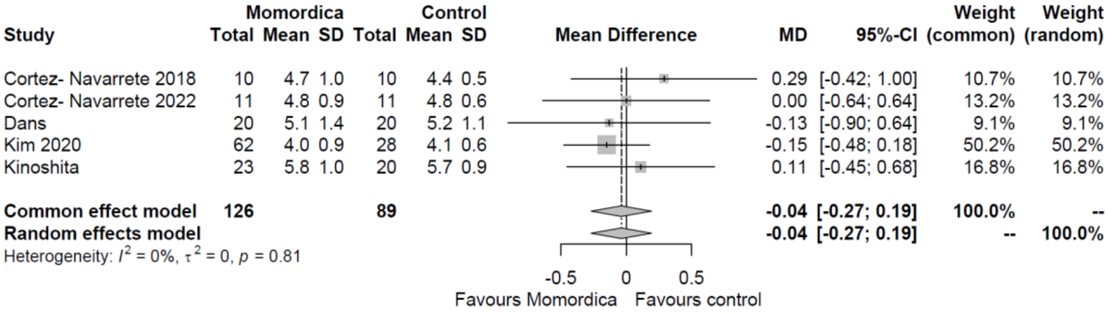
Study
Momordica Total Mean SD
Control Total Mean SD
Mean Difference
MD 95%-CI
Weight (common)
Weight (random)
Cortez- Navarrete 2018 10 4.7 1.0 10 4.4 0.5 0.29 [-0.42; 1.00] 10.7% 10.7%
Cortez- Navarrete 2022 11 4.8 0.9 11 4.8 0.6 0.00 [-0.64; 0.64] 13.2% 13.2%
Dans 20 5.1 1.4 20 5.2 1.1 -0.13 [-0.90; 0.64] 9.1% 9.1%
Kim 2020 62 4.0 0.9 28 4.1 0.6 -0.15 [-0.48; 0.18] 50.2% 50.2%
Kinoshita 23 5.8 1.0 20 5.7 0.9 0.11 [-0.45; 0.68] 16.8% 16.8%
Common effect model 126 89 -0.04 [-0.27; 0.19] 100.0% --
Random effects model -0.04 [-0.27; 0.19] -- 100.0%
Heterogeneity: I2 = 0%, τ2 = 0, p = 0.81
-0.5 0 0.5
Favours Momordica Favours control

D

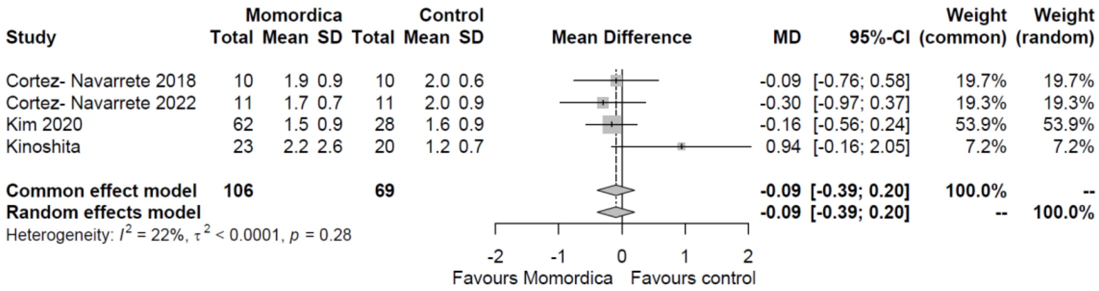

Supplementary Figure 3S. The effect of *Momordica charantia* on HDL (**A**), LDL (**B**), total cholesterol levels (**C**), and triglyceride levels (**D**) compared to placebo in the meta-analyses of the post-intervention values using the random effects and common effect models

A

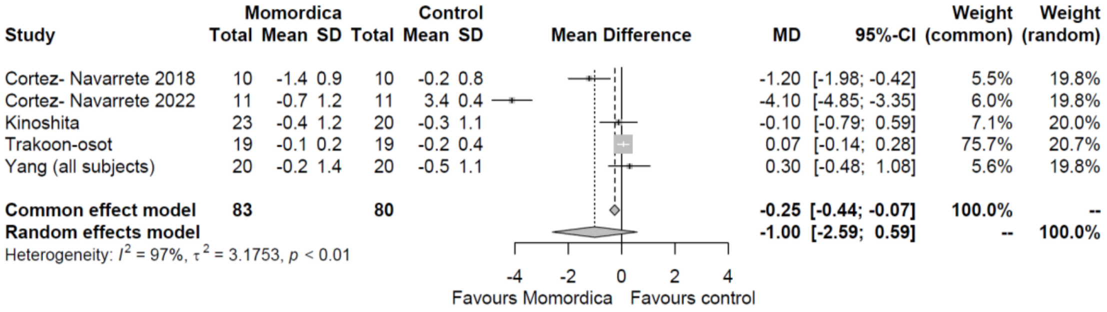
Momordica
Control
Study
Total Mean SD Total Mean SD
Mean Difference
MD
95%-CI
Weight (common)
Weight (random)
Cortez- Navarrete 2018 10 -1.4 0.9 10 -0.2 0.8 -1.20 [-1.98; -0.42] 5.5% 19.8%
Cortez- Navarrete 2022 11 -0.7 1.2 11 3.4 0.4 -4.10 [-4.85; -3.35] 6.0% 19.8%
Kinoshita 23 -0.4 1.2 20 -0.3 1.1 -0.10 [-0.79; 0.59] 7.1% 20.0%
Trakoon-osot 19 -0.1 0.2 19 -0.2 0.4 0.07 [-0.14; 0.28] 75.7% 20.7%
Yang (all subjects) 20 -0.2 1.4 20 -0.5 1.1 0.30 [-0.48; 1.08] 5.6% 19.8%
Common effect model 83 80 -0.25 [-0.44; -0.07] 100.0% --
Random effects model -1.00 [-2.59; 0.59] -- 100.0%
Heterogeneity: I^2 = 97%, τ^2 = 3.1753, p < 0.01
-4 -2 0 2 4
Favours Momordica Favours control

B

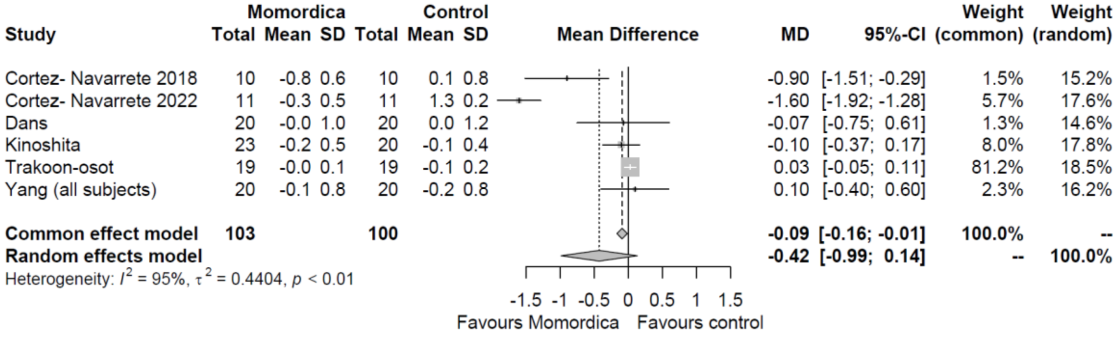
Momordica
Control
Study
Total Mean SD Total Mean SD
Mean Difference
MD
95%-CI
Weight (common)
Weight (random)
Cortez- Navarrete 2018 10 -0.8 0.6 10 0.1 0.8 -0.90 [-1.51; -0.29] 1.5% 15.2%
Cortez- Navarrete 2022 11 -0.3 0.5 11 1.3 0.2 -1.60 [-1.92; -1.28] 5.7% 17.6%
Dans 20 -0.0 1.0 20 0.0 1.2 -0.07 [-0.75; 0.61] 1.3% 14.6%
Kinoshita 23 -0.2 0.5 20 -0.1 0.4 -0.10 [-0.37; 0.17] 8.0% 17.8%
Trakoon-osot 19 -0.0 0.1 19 -0.1 0.2 0.03 [-0.05; 0.11] 81.2% 18.5%
Yang (all subjects) 20 -0.1 0.8 20 -0.2 0.8 0.10 [-0.40; 0.60] 2.3% 16.2%
Common effect model 103 100 -0.09 [-0.16; -0.01] 100.0% --
Random effects model -0.42 [-0.99; 0.14] -- 100.0%
Heterogeneity: I^2 = 95%, τ^2 = 0.4404, p < 0.01
-1.5 -1 -0.5 0 0.5 1 1.5
Favours Momordica Favours control

C

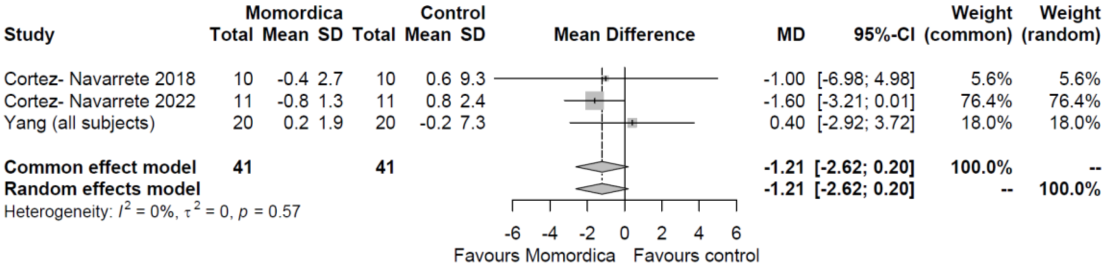
Momordica
Control
Study
Total Mean SD Total Mean SD
Mean Difference
MD
95%-CI
Weight (common)
Weight (random)
Cortez- Navarrete 2018 10 -0.4 2.7 10 0.6 9.3 -1.00 [-6.98; 4.98] 5.6% 5.6%
Cortez- Navarrete 2022 11 -0.8 1.3 11 0.8 2.4 -1.60 [-3.21; 0.01] 76.4% 76.4%
Yang (all subjects) 20 0.2 1.9 20 -0.2 7.3 0.40 [-2.92; 3.72] 18.0% 18.0%
Common effect model 41 41 -1.21 [-2.62; 0.20] 100.0% --
Random effects model -1.21 [-2.62; 0.20] -- 100.0%
Heterogeneity: I^2 = 0%, τ^2 = 0, p = 0.57
-6 -4 -2 0 2 4 6
Favours Momordica Favours control

D

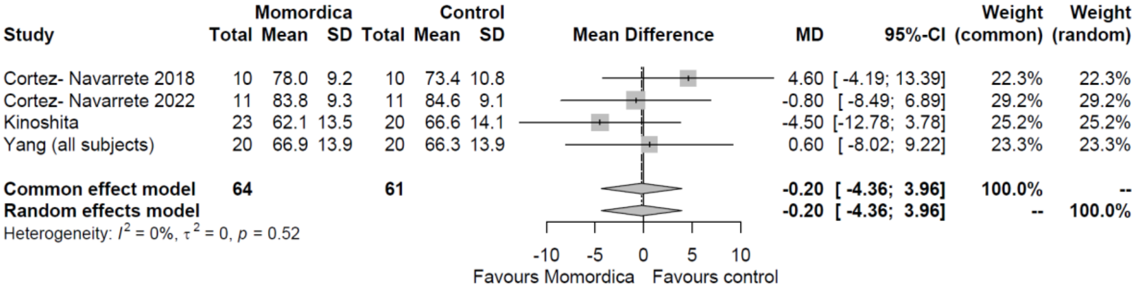
Momordica
Control
Study
Total Mean SD Total Mean SD
Mean Difference
MD
95%-CI
Weight (common)
Weight (random)
Cortez- Navarrete 2018 10 78.0 9.2 10 73.4 10.8 4.60 [-4.19; 13.39] 22.3% 22.3%
Cortez- Navarrete 2022 11 83.8 9.3 11 84.6 9.1 -0.80 [-8.49; 6.89] 29.2% 29.2%
Kinoshita 23 62.1 13.5 20 66.6 14.1 -4.50 [-12.78; 3.78] 25.2% 25.2%
Yang (all subjects) 20 66.9 13.9 20 66.3 13.9 0.60 [-8.02; 9.22] 23.3% 23.3%
Common effect model 64 61 -0.20 [-4.36; 3.96] 100.0% --
Random effects model -0.20 [-4.36; 3.96] -- 100.0%
Heterogeneity: I^2 = 0%, τ^2 = 0, p = 0.52
-10 -5 0 5 10
Favours Momordica Favours control

E

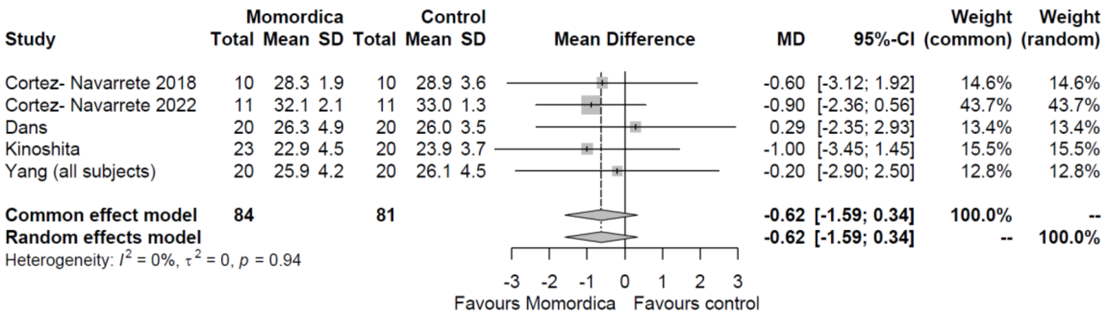

F

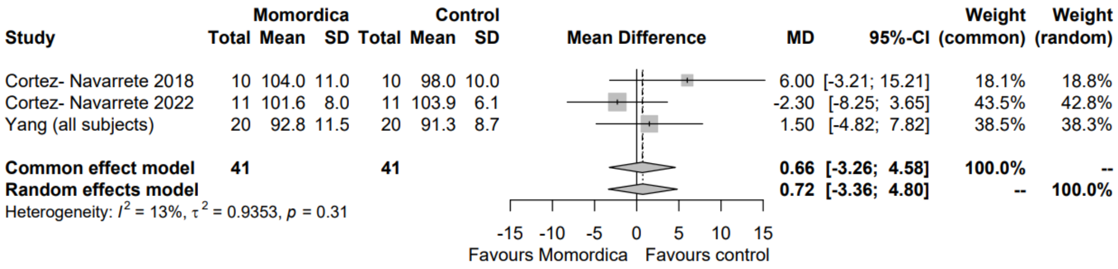

Supplementary Figure 4S. The effect of *Momordica charantia* on body weight, BMI and body fat compared to placebo in the meta-analyses of the change scores (A, B and C respectively) and the post-intervention body weight, BMI and waist circumference values (D, E and F, respectively) using the random effects and common effect models

A

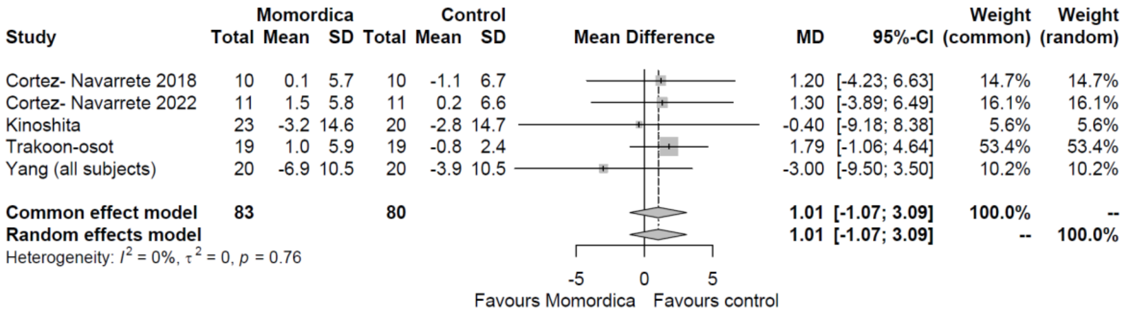

B

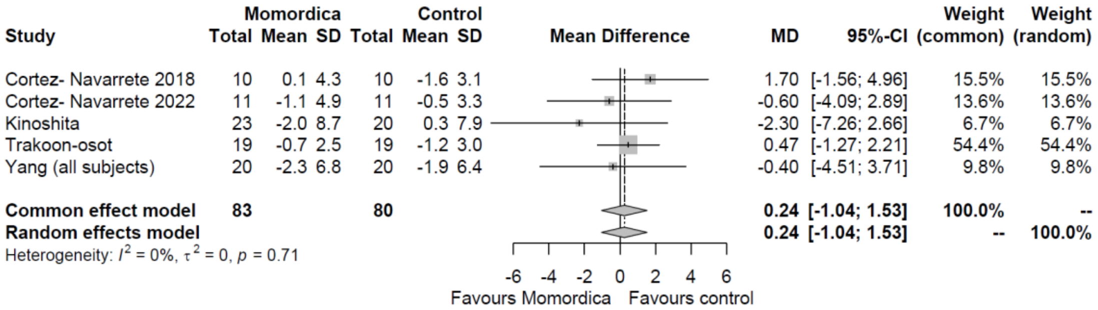

C

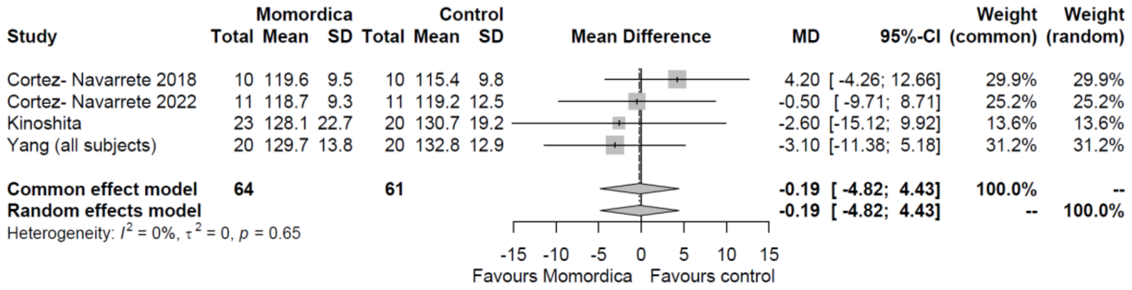

D

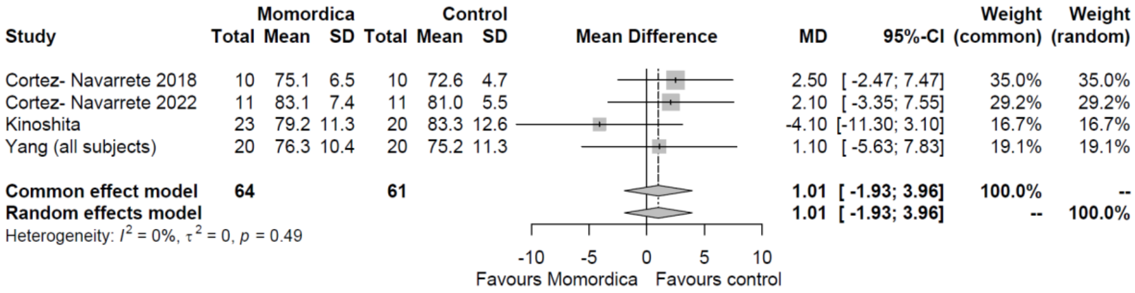

Supplementary Figure 5S. The effect of *Momordica charantia* on systolic and diastolic blood pressure compared to placebo in the meta-analyses of the change scores (A and B, respectively) and the post-intervention values (C and D, respectively) using the random effects and common effect models

A

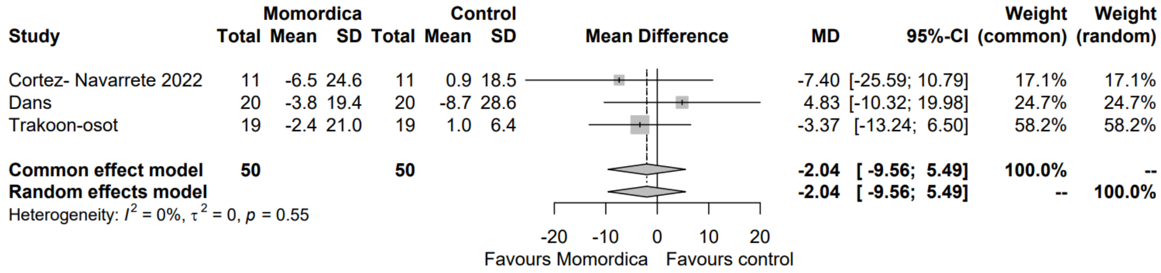
Momordica
Control
Study
Total Mean SD Total Mean SD
Mean Difference
MD 95%-CI
Weight (common)
Weight (random)
Cortez- Navarrete 2022 11 -6.5 24.6 11 0.9 18.5 -7.40 [-25.59; 10.79] 17.1% 17.1%
Dans 20 -3.8 19.4 20 -8.7 28.6 4.83 [-10.32; 19.98] 24.7% 24.7%
Trakoon-osot 19 -2.4 21.0 19 1.0 6.4 -3.37 [-13.24; 6.50] 58.2% 58.2%
Common effect model 50 50 -2.04 [-9.56; 5.49] 100.0% --
Random effects model -2.04 [-9.56; 5.49] -- 100.0%
Heterogeneity: I2 = 0%, τ2 = 0, p = 0.55
-20 -10 0 10 20
Favours Momordica Favours control

B

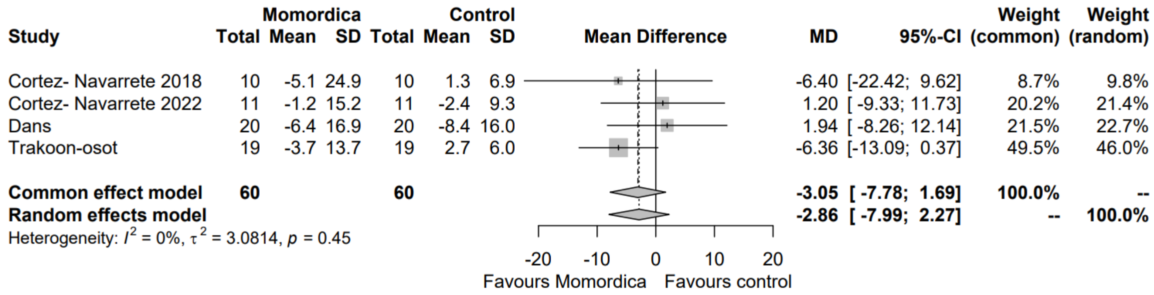
Momordica
Control
Study
Total Mean SD Total Mean SD
Mean Difference
MD 95%-CI
Weight (common)
Weight (random)
Cortez- Navarrete 2018 10 -5.1 24.9 10 1.3 6.9 -6.40 [-22.42; 9.62] 8.7% 9.8%
Cortez- Navarrete 2022 11 -1.2 15.2 11 -2.4 9.3 1.20 [-9.33; 11.73] 20.2% 21.4%
Dans 20 -6.4 16.9 20 -8.4 16.0 1.94 [-8.26; 12.14] 21.5% 22.7%
Trakoon-osot 19 -3.7 13.7 19 2.7 6.0 -6.36 [-13.09; 0.37] 49.5% 46.0%
Common effect model 60 60 -3.05 [-7.78; 1.69] 100.0% --
Random effects model -2.86 [-7.99; 2.27] -- 100.0%
Heterogeneity: I2 = 0%, τ2 = 3.0814, p = 0.45
-20 -10 0 10 20
Favours Momordica Favours control

C

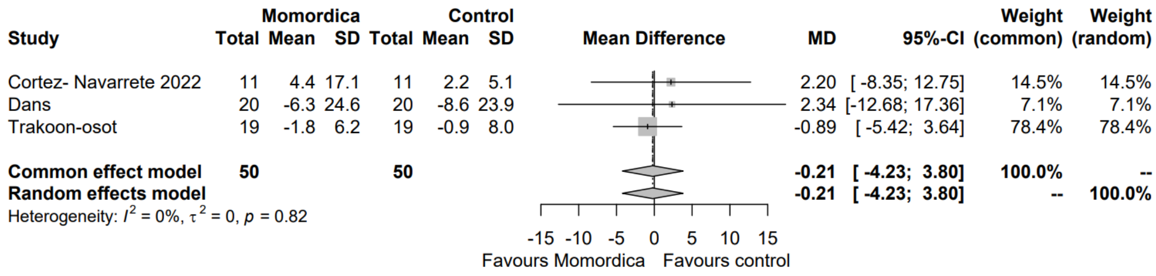
Momordica
Control
Study
Total Mean SD Total Mean SD
Mean Difference
MD 95%-CI
Weight (common)
Weight (random)
Cortez- Navarrete 2022 11 4.4 17.1 11 2.2 5.1 2.20 [-8.35; 12.75] 14.5% 14.5%
Dans 20 -6.3 24.6 20 -8.6 23.9 2.34 [-12.68; 17.36] 7.1% 7.1%
Trakoon-osot 19 -1.8 6.2 19 -0.9 8.0 -0.89 [-5.42; 3.64] 78.4% 78.4%
Common effect model 50 50 -0.21 [-4.23; 3.80] 100.0% --
Random effects model -0.21 [-4.23; 3.80] -- 100.0%
Heterogeneity: I2 = 0%, τ2 = 0, p = 0.82
-15 -10 -5 0 5 10 15
Favours Momordica Favours control

D

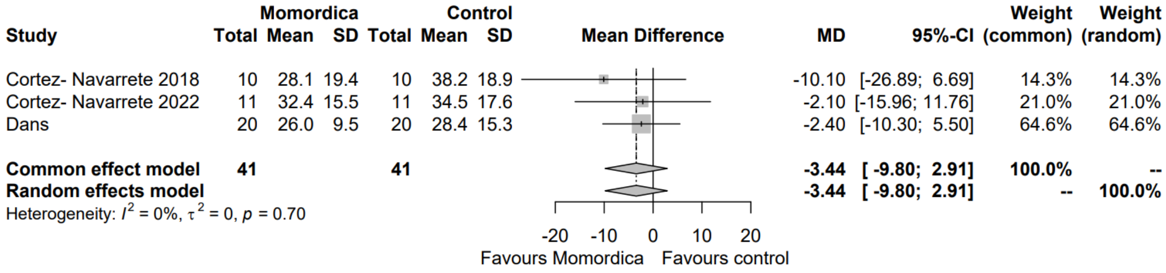
Momordica
Control
Study
Total Mean SD Total Mean SD
Mean Difference
MD 95%-CI
Weight (common)
Weight (random)
Cortez- Navarrete 2018 10 28.1 19.4 10 38.2 18.9 -10.10 [-26.89; 6.69] 14.3% 14.3%
Cortez- Navarrete 2022 11 32.4 15.5 11 34.5 17.6 -2.10 [-15.96; 11.76] 21.0% 21.0%
Dans 20 26.0 9.5 20 28.4 15.3 -2.40 [-10.30; 5.50] 64.6% 64.6%
Common effect model 41 41 -3.44 [-9.80; 2.91] 100.0% --
Random effects model -3.44 [-9.80; 2.91] -- 100.0%
Heterogeneity: I2 = 0%, τ2 = 0, p = 0.70
-20 -10 0 10 20
Favours Momordica Favours control

E

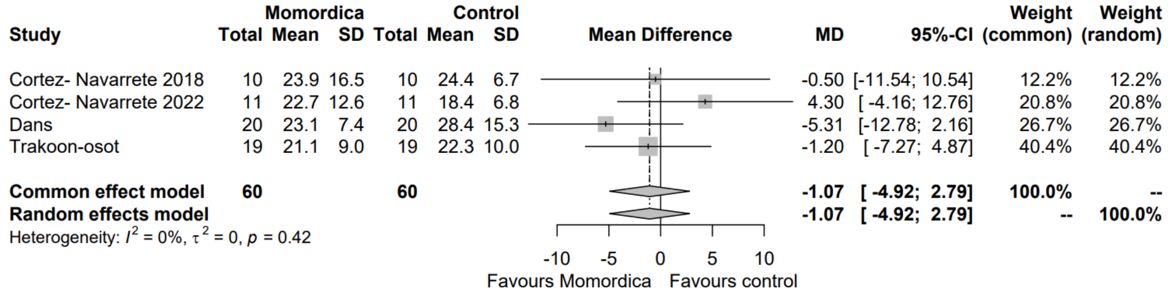

F

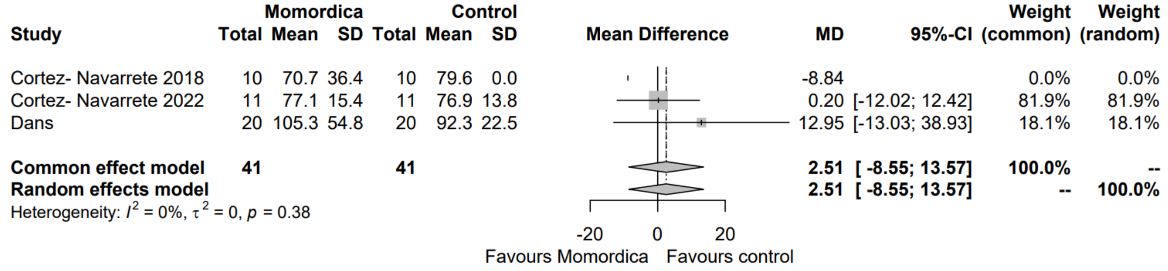

Supplementary Figure 6S. The effect of *Momordica charantia* on ALT, AST and creatinine levels compared to placebo in the meta-analyses of the change scores (**A, B** and **C**, respectively) and the post-intervention values (**D, E** and **F**, respectively) using the random effects and common effect models
